# Impact of Diabetes on Management and Outcomes in Patients with Borderline FFR_CT_

**DOI:** 10.3390/jcdd13010011

**Published:** 2025-12-24

**Authors:** Yanchun Chen, Zhan Feng, Wenjing Jia, Xiaoyu Ma, Zhengjie He, Hui Lou, Hongjie Hu, Zhen Zhou, Lei Xu

**Affiliations:** 1 Department of Radiology, Beijing Anzhen Hospital, Capital Medical University, No. 2 Anzhen Rd, Chaoyang District, Beijing 100020, China; 18910058705@163.com (Y.C.); jiawj12@163.com (W.J.); xiaoyuma001@163.com (X.M.); 2 Department of Radiology, The First Affiliated Hospital of Zhejiang University, Zhejiang University School of Medicine, 79 Qingchun Road, Hangzhou 310010, China; gerxyuan@zju.edu.cn; 3 Department of Radiology, Sir Run Run Shaw Hospital, Zhejiang University School of Medicine, 3 East Qingchun Road, Hangzhou 310010, China; 19548177487@163.com (Z.H.);; 4 Department of Radiology, Zhejiang University School of Medicine, The First Affiliated Hospital of Zhejiang University, 79 Qingchun Road, Hangzhou 310010, China; gtdtftltytdlh7@163.com

**Keywords:** diabetes, FFR_CT_, ICA, revascularization, MACE

## Abstract

**Background:** The impact of diabetes on the management and outcomes of patients with borderline CT-derived fractional flow reserve (FFR_CT_) remains unclear. **Methods:** This multicenter study enrolled symptomatic patients with suspected coronary artery disease who underwent Coronary computed tomography angiography (CCTA) between June 2021 and May 2023, yielding FFR_CT_ values between 0.70 and 0.80. Revascularization occurring within 90 days after CCTA was documented. The endpoint was major adverse cardiovascular events (MACE), as a composite of all-cause death, nonfatal myocardial infarction, and unplanned revascularization. Outcomes were analyzed using Cox proportional hazards models, while the relationship between FFR_CT_ and MACE was examined using restricted cubic spline analysis (RCS). **Results:** This analysis included 1515 patients with borderline FFR_CT_ values, comprising 503 (33.2%) with diabetes. Over a median follow-up of 985 days, 117 MACE occurred. Multivariate analysis showed that revascularization was independently associated with a reduced risk of the endpoint, a protective effect consistent in both non-diabetic (adjusted HR [aHR] 0.53, 95% CI 0.29–0.96; *p* = 0.036) and diabetic patients (aHR 0.25, 95% CI 0.09–0.71; *p* = 0.009). RCS revealed a significant non-linear relationship between FFR_CT_ and MACE in non-diabetic patients (*p* = 0.002). **Conclusions:** In patients with borderline FFR_CT_, revascularization was linked to a lower incidence of MACE, and this association was consistent regardless of diabetes status.

## 1. Introduction

Diabetes mellitus (DM) is a well-established risk factor for major adverse cardiovascular events (MACE) [1], as highlighted in both AHA/ASA and ESC guidelines [2,3]. Appropriate coronary artery disease (CAD) risk stratification and multifactorial risk management are essential for cardiovascular prevention in DM [4]. Coronary CT angiography (CCTA) has become the first-line diagnostic tool for CAD, providing a comprehensive evaluation of both anatomic and functional severity [5]. Computed tomography-derived fractional flow reserve (FFR_CT_) is a noninvasive method for assessing hemodynamically significant coronary stenosis based on CCTA [6]. This innovative technique assists in guiding optimal treatment strategies and reducing unnecessary invasive procedures [7,8]. Current guidelines and expert consensus suggest that FFR_CT_ values >0.80 generally exclude flow-limiting disease and justify conservative management, whereas values <0.70 indicate hemodynamically significant stenosis that warrants revascularization [9,10]. Borderline FFR_CT_ values (0.70–0.80) pose diagnostic and management challenges, with interpretation and treatment strategies remaining ambiguous [11,12]. In diabetic patients, this diagnostic uncertainty is further complicated by the interplay between coronary pathophysiology and metabolic dysregulation, potentially influencing outcomes.

In a prespecified post hoc analysis of the prospective PREVENT trial, preventive percutaneous coronary intervention (PCI) for non-flow-limiting vulnerable plaques (fractional flow reserve >0.80) reduced the primary endpoint at 2 years compared with optimal medical therapy (OMT) alone, with no significant difference between diabetic and non-diabetic patients [13]. Expanding on the role of FFR_CT_ in diabetes, it is important to recognize that CAD in this population often involves more diffuse atherosclerosis, microvascular dysfunction, and endothelial abnormalities. These factors can alter the relationship between anatomical stenosis and its functional significance, making functional assessments such as FFR_CT_ particularly valuable. Evidence indicates that FFR_CT_ serves as an independent predictor of MACE in diabetic patients, offering additive prognostic value beyond conventional clinical and imaging parameters. However, its predictive accuracy diminishes when FFR_CT_ values fall within the intermediate range of 0.70–0.80 [10,14]. Current evidence is insufficient to determine how diabetes mellitus affects management and outcomes in patients with borderline FFR_CT_ values. Therefore, this study aimed to evaluate how diabetes influences clinical decision pathways in patients with borderline FFR_CT_ values, particularly the utilization of invasive angiography and revascularization, and to assess its impact on long-term outcomes, focusing on the risk stratification potential of borderline FFR_CT_ values for predicting cardiovascular events.

## 2. Methods

### 2.1. Patients Population

**Figure 1:**
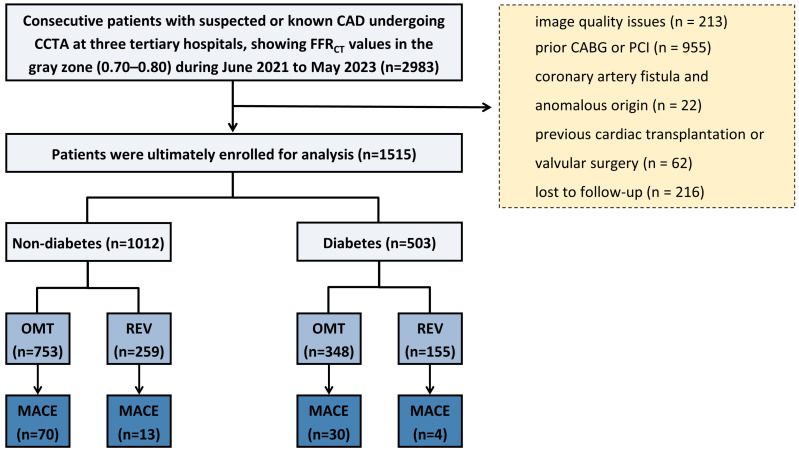
**Flowchart**. CAD = coronary artery disease; CCTA = coronary computed tomography angiography; FFR_CT_ = coronary CT angiography-derived fractional flow reserve; PCI = percutaneous coronary intervention; CABG = coronary artery bypass grafting; OMT = optimal medical therapy; REV = revascularization; MACE = major adverse cardiovascular events.

The study flowchart is shown in Figure 1. Briefly, symptomatic patients with suspected CAD were enrolled from three tertiary hospitals in China between June 2021 and May 2023. All patients first underwent CCTA, which demonstrated at least one coronary lesion in a major vessel (left main coronary artery, left anterior descending artery, left circumflex artery, or right coronary artery). Among these, patients were included for further analysis if clinically indicated FFR_CT_ revealed values between 0.70 and 0.80 in the affected vessel. Referral for FFR_CT_ was determined by factors such as the clinical likelihood of ischemia, resource availability, local expertise, patient characteristics, and patient preference [15]. Patients were excluded for the following reasons: poor image quality or failure of FFR_CT_ analysis, a history of coronary revascularization (PCI or coronary artery bypass grafting [CABG]) prior to CCTA, anomalous coronary artery origin or coronary artery fistula, previous cardiac transplantation or valvular surgery, or loss to follow-up. Baseline demographic characteristics, cardiovascular risk factors, and relevant laboratory data (including fasting blood glucose and HbA1c) were collected. DM was diagnosed based on any of the following criteria: a documented history of diabetes in the medical records, an HbA1c level of ≥6.5% at admission, or current use of antidiabetic medication [16]. However, relevant biochemical test results were not available for all participants. The proportion of missing data was reported. A sensitivity analysis was conducted in the cohort with complete data to assess robustness. This retrospective study was approved by the institutional review boards of all participating centers. The requirement for written informed consent was waived in accordance with the Declaration of Helsinki.

### 2.2. CCTA Acquisition

Details of the CCTA protocols are provided in the Supplemental Methods. Image quality was assessed on a per-patient basis using a 5-point scale, as previously described [17]. Patients with image quality scores of 1 or 2 were excluded. CT images were initially interpreted by local investigators at each participating center according to the Coronary Artery Disease Reporting and Data System (CAD-RADS) version 2.0 [18] and subsequently reviewed by senior readers. The coronary artery calcium score (CACS) was calculated from baseline noncontrast CT scans using the Agatston method [19] and categorized as 0 (none), 1–100 (mild), 101–300 (moderate), or >300 (severe), in line with current guidelines and expert consensus [20].

### 2.3. FFR_CT_ Analysis

FFR_CT_ values were computationally derived from standard CCTA images using an on-site, machine learning-based approach. All major epicardial coronary arteries with a lumen diameter ≥2.5 mm were included in the analysis. The assessments were performed on the DEEPVESSEL FFR workstation (Keya Medical Technology Co., Ltd., Beijing, China), an artificial intelligence (AI) platform that applies validated algorithms to simulate FFR values directly from CCTA data, as previously described [21]. DEEPVESSEL FFR has received 510 (k) clearance from the U.S. Food and Drug Administration, and prior studies have confirmed its diagnostic accuracy [22]. FFR_CT_ values were recorded for each coronary vessel. In cases involving multiple stenoses, the FFR_CT_ value distal to the most severe lesion was used. Lesion-specific FFR_CT_ was defined as the simulated FFR value measured 20 mm distal to the lesion of interest. For per-patient analysis, the lowest FFR_CT_ value among all vessels was selected for prognostic evaluation.

### 2.4. Management Strategy and Clinical Endpoints

The post-CCTA treatment strategy was determined at the discretion of the referring physician, who had access to the CCTA findings, FFR_CT_ results, and other relevant clinical information. The index treatment strategies in real-world practice, including invasive coronary angiography (ICA), revascularization (PCI or CABG), and OMT within 90 days, were recorded. The primary endpoint was MACE, defined as a composite of all-cause mortality, nonfatal myocardial infarction (MI), and unplanned coronary revascularization [23]. The secondary endpoint was a composite of all-cause mortality and nonfatal MI. Nonfatal MI was diagnosed by a characteristic rise and/or fall in cardiac biomarkers (preferably troponin) above the 99th percentile upper reference limit, plus at least one of the following: ischemic symptoms, new ECG changes (pathological Q waves or ST elevation), or angiographic evidence of CAD [24]. Unplanned revascularization was defined as PCI or CABG that met both of the following criteria: (1) the procedure was unscheduled, meaning it was neither performed as part of routine care during the initial procedure nor preplanned within 90 days (or per institutional standards); and (2) it was ischemia-driven, prompted by objective clinical evidence of ischemia such as hospitalization for recurrent angina or documented ischemic changes on ECG or noninvasive imaging, ultimately leading to revascularization [23,25]. Patient follow-up was performed through telephone interviews and review of medical records. The primary endpoint was the composite outcome, with time to event defined as the period from baseline CCTA to the first qualifying event. All potential endpoint events identified during follow-up were independently adjudicated by a dedicated clinical events committee. The committee members, blinded to treatment allocation, reviewed all relevant source documentation against pre-specified, standardized criteria to confirm both the occurrence and classification of each event. Patients without an endpoint event were censored at the date of their last follow-up contact. 

### 2.5. Statistical Analysis

Continuous variables were expressed as mean ± standard deviation or median with interquartile range (IQR), depending on the normality of distribution. Categorical variables were presented as counts with corresponding percentages. The Kolmogorov–Smirnov test was used to assess the normality of continuous variables. Differences between groups were analyzed using independent t-tests for normally distributed data and the Mann–Whitney U test for non-normally distributed data. Pearson’s chi-square test was applied for categorical variables. A one-way analysis of variance (ANOVA) was used to compare ICA stenosis severity across the four groups stratified by diabetes mellitus status and revascularization. Post hoc pairwise comparisons between groups were conducted, and mean differences with their 95% confidence intervals (CIs) were reported. For outcome analysis, cumulative event incidence was estimated using the log-rank test. Univariable and multivariable Cox proportional hazards models were used to estimate hazard ratios (HRs) with 95% CIs. Variables with *p* ≤ 0.10 in univariate analysis were included in the multivariate model. FFR_CT_ values were multiplied by 100 for clearer clinical and statistical interpretation. The nonlinear relationship between FFR_CT_ value and MACE was evaluated separately in diabetic and non-diabetic groups using restricted cubic splines (RCS). Subgroup analyses were conducted according to stenosis severity (CAD-RADS 2.0), CACS (<300 vs. ≥300), and CT-FFR values (≤0.75 vs. >0.75), based on thresholds established in previous studies [18,20]. To mitigate selection bias arising from the non-randomized, clinician-driven decision for revascularization, we performed a propensity score matching (PSM) analysis. Propensity scores were estimated using a multivariable logistic regression model that incorporated all available baseline characteristics. A 1:2 nearest-neighbor matching algorithm was employed with a caliper width set to 0.25 standard deviations of the logit of the propensity score. Covariate balance between the revascularized and non-revascularized groups before and after matching was rigorously assessed using standardized mean differences (SMDs), where an absolute SMD < 0.1 was considered indicative of satisfactory balance. Comparative analyses of clinical outcomes were subsequently conducted on the matched cohort. To test the robustness of our findings against the potential influence of very early events, we performed a sensitivity analysis by excluding all patients who experienced an unplanned revascularization within 30 days of the initial procedure. A two-sided *p*-value < 0.05 was considered statistically significant. All analyses were performed using R version 4.1.0.

## 3. Results

### 3.1. Study Population and Baseline Characteristics

Of the initial 2983 enrolled patients, 1468 were excluded due to poor image quality (n = 213), prior CABG or PCI (n = 955), coronary artery fistula or anomalous origin (n = 22), previous cardiac transplantation or valvular surgery (n = 62), and loss to follow-up (n = 216). The final analysis included 1515 patients with FFR_CT_ values in the gray zone (0.70–0.80), comprising 503 (33.2%) diabetic and 1012 (66.8%) non-diabetic individuals (Figure 1). As shown in Table 1, diabetic patients were significantly older (63 ± 9 vs. 62 ± 10 years, *p* = 0.009), with lower male predominance (64.2% vs. 69.6%, *p* = 0.036) and a higher prevalence of hypertension (70.4% vs. 58.5%, *p* < 0.001), hyperlipidemia (69.0% vs. 61.6%, *p* = 0.005), and family history of cardiovascular disease (36.0% vs. 27.4%, *p* < 0.001). Metabolic assessment showed substantially higher fasting glucose and HbA1c levels in diabetic patients (both *p* < 0.001). Imaging parameters demonstrated higher CACS in diabetics (534 [IQR: 171–1099] vs. 298 [IQR: 82–790] Agatston units, *p* < 0.001) and marginally lower FFR_ct_ values (0.758 ± 0.026 vs. 0.762 ± 0.024, *p* = 0.016). No significant differences were observed in smoking status, follow-up duration, vessel distribution, or CAD-RADS classification (all *p* > 0.05).

### 3.2. Interventional Management Stratified by Diabetes

**Figure 2:**
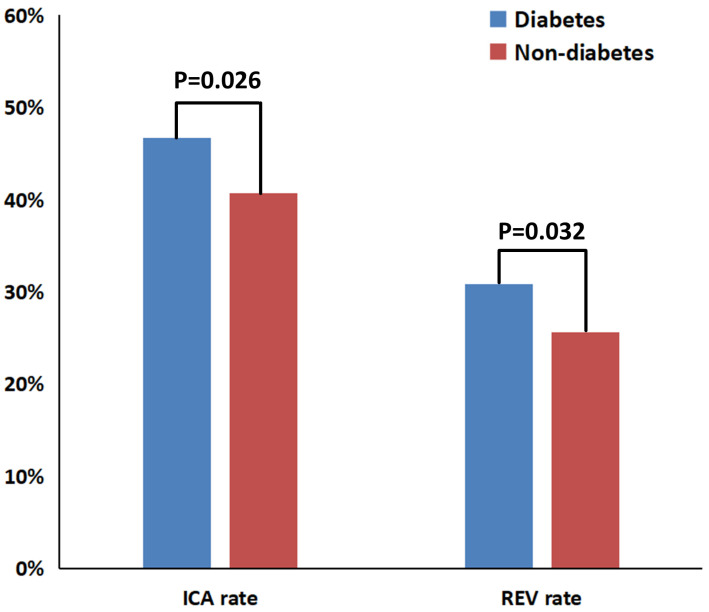
Bar chart comparing ICA and revascularization rates between diabetic and non-diabetic groups. ICA = invasive coronary angiography; REV = revascularization.

The heatmap visualization (Figure S1) revealed distinct clinical management patterns between study groups. Among the total cohort, 235 diabetic patients (15.5%) underwent ICA, whereas 268 (17.6%) were managed conservatively. Revascularization procedures were performed in 155 diabetic individuals (10.2%), while 348 (23.0%) received OMT alone. Comparative analyses of participants stratified by diabetes and revascularization status are presented in Table S1. Both diabetic and non-diabetic revascularized groups exhibited markedly higher CAD-RADS 4 prevalence and significantly lower FFR_CT_ values compared with medically managed groups (all *p* < 0.001). Diabetic patients had a significantly higher prevalence of ICA (46.7% vs. 40.7%, *p* = 0.026) and revascularization (30.8% vs. 25.6%, *p* = 0.032) compared with non-diabetic patients (Figure 2).

Figure S2 details ICA stenosis rates among the four subgroups stratified by diabetes and revascularization status. One-way ANOVA showed significant overall differences (*p* < 0.001). Post hoc pairwise comparisons indicated that revascularization was associated with significantly higher stenosis rates in both non-diabetic (mean difference: −20.66, 95% CI: −23.91 to −17.41; *p* < 0.001) and diabetic patients (mean difference: −20.61, 95% CI: −24.98 to −16.24; *p* < 0.001). In contrast, diabetes status itself did not significantly affect stenosis rates, regardless of revascularization status (all *p* > 0.05).

### 3.3. Study Outcomes

During a median follow-up of 985 days (IQR: 504–1147), MACE occurred in 117 patients (5.9%), including unplanned revascularization in 89 (5.9%), all-cause death in 27 (1.8%), and non-fatal MI in 10 (0.7%). There were eight instances of unplanned revascularization following non-fatal MI, and one case of death following unplanned revascularization. Comparative analysis of outcomes by diabetes status is shown in Table 2. Revascularization was consistently associated with a significantly lower risk of MACE compared with OMT alone, both in non-diabetic (5.0% vs. 9.3%, *p* = 0.030) and diabetic patients (2.6% vs. 8.6%, *p* = 0.013). This benefit was primarily due to a marked reduction in unplanned revascularization procedures within the revascularization groups, evident in both non-diabetic (3.1% vs. 6.9%, *p* = 0.025) and diabetic patients (1.3% vs. 7.8%, *p* = 0.004). However, the risks of all-cause death, non-fatal MI, and the composite secondary outcome did not differ significantly between revascularization and OMT strategies.

**Figure 3:**
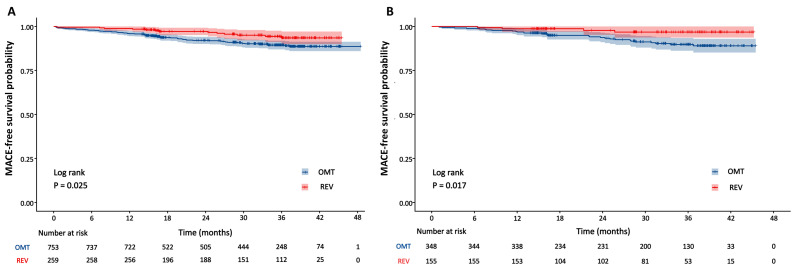
Kaplan–Meier curves for MACE by revascularization status in non-diabetic and diabetic groups. (**A**) Non-diabetic group; (**B**) diabetic group. OMT = optimal medical therapy; REV = revascularization.

Cox proportional hazards analysis for MACE by diabetes status is presented in Table 3. After multivariate adjustment for potential confounders, revascularization remained independently associated with a significantly reduced risk of the primary endpoint. This protective effect persisted in both non-diabetic (adjusted HR 0.53, 95% CI 0.29–0.96; *p* = 0.036) and diabetic patients (aHR 0.25, 95% CI 0.09–0.71; *p* = 0.009). In the diabetic cohort, lower FFR_CT_ values were independently associated with an increased risk of MACE (aHR 0.87, 95% CI 0.77–0.99; *p* = 0.031). No other variables independently predicted outcomes. Kaplan–Meier curves for the primary endpoint, stratified by revascularization status, are presented separately for non-diabetic and diabetic cohorts in Figure 3. The log-rank test indicated significantly higher event-free survival among revascularized patients, both in the non-diabetic (*p* = 0.025) and diabetic cohorts (*p* = 0.017). Cause-specific survival analysis for hard events is now presented in Figure S3, which shows no significant difference between revascularized and non-revascularized patients in either cohort (all *p* > 0.05). In contrast, revascularized patients showed significantly lower rates of unplanned revascularization in both the diabetic (*p* = 0.006) and non-diabetic (*p* = 0.021) cohorts.

### 3.4. Exploration of Association Shape and Effect Consistency

Restricted cubic spline analysis revealed a significant non-linear relationship between FFR_CT_ values and MACE hazard in non-diabetic patients (*p* for non-linearity = 0.002) (Figure S4). The fitted curve displayed a distinct inverted U-shape, with the HR equal to 1 at an FFR_CT_ value of 0.77. In contrast, no significant non-linear association was detected in diabetic patients (*p* for non-linearity = 0.147).

**Figure 4:**
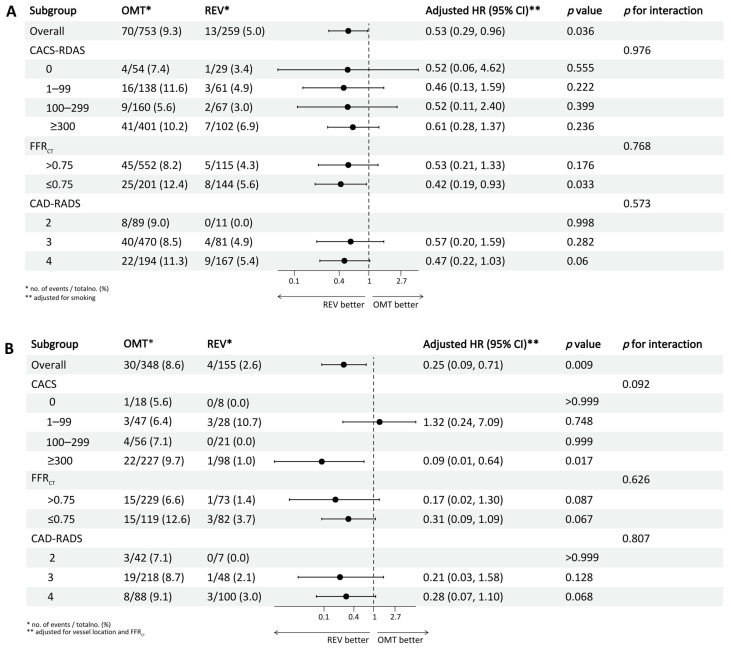
Forest plot showing subgroup analyses of the association between revascularization and MACE. (**A**) Non-diabetic group; (**B**) diabetic group. OMT = optimal medical therapy; REV = revascularization; HR = hazard ratio; CACS = coronary artery calcium score; FFR_CT_ = coronary CT angiography-derived fractional flow reserve; CAD-RADS = coronary artery disease reporting and data system.

Subgroup analyses stratified by CACS, CAD-RADS, and FFR_CT_ thresholds (≤0.75) within diabetic and non-diabetic cohorts revealed no significant interaction effects (all *p* for interaction > 0.05) (Figure 4). Among non-diabetic patients, revascularization was associated with a significantly lower MACE risk in those with FFR_CT_ ≤ 0.75 (HR 0.42, 95% CI 0.19–0.93; *p* = 0.033). In diabetic patients, a significant treatment benefit was observed only in the subgroup with CACS ≥ 300 (HR 0.09, 95% CI 0.01–0.64; *p* = 0.017), with no significant risk reduction in other subgroups (all *p* > 0.05). A 1:2 PSM analysis, incorporating all baseline characteristics, was conducted to address potential confounding. This yielded a well-balanced matched cohort of 388 revascularized and 587 conservatively managed patients. Covariate balance was excellent post-matching (Figure S5). In this matched cohort, revascularization remained associated with a significantly lower risk of the MACE compared to conservative management (*p* = 0.001) (Figure S6). After excluding four patients who underwent unplanned revascularization within 30 days, Kaplan–Meier analysis showed that the association between revascularization and reduced risk of the primary composite endpoint remained significant (*p* = 0.017) (Figure S7).

Sensitivity analysis was conducted in a subgroup of 569 (37.6%) patients with complete fasting glucose and HbA1c data to confirm the robustness of the primary findings (Table S2). Consistent with the main analysis, revascularization remained a significant independent predictor of improved MACE outcomes, irrespective of diabetes status. Among non-diabetic patients, revascularization was associated with a 67% risk reduction (aHR 0.33, 95% CI 0.12–0.91; *p* = 0.032), while diabetic patients experienced an even greater 74% reduction (aHR 0.26, 95% CI 0.07–0.89; *p* = 0.032).

## 4. Discussion

This study investigated the impact of diabetes on treatment strategies and clinical outcomes in patients with borderline FFR_CT_ values. The findings revealed that: first, within this gray-zone range, diabetic patients underwent significantly more interventional strategies (including ICA examination and revascularization) after CCTA than non-diabetic patients; second, after multivariable adjustment, revascularization was significantly associated with a reduced risk of MACE, a benefit primarily driven by a decrease in unplanned revascularizations, with no significant association observed for hard endpoints; and finally, Cox multivariable analysis demonstrated that FFR_CT_ value served as an independent predictor of MACE in the diabetic population, whereas a nonlinear relationship was observed between FFR_CT_ values and MACE in non-diabetic patients.

Diabetic patients underwent significantly more revascularization procedures in our study, consistent with current guideline recommendations and the long-term outcomes of the FREEDOM trial, which support more aggressive intervention in this high-risk population [26]. Regarding clinical outcomes, the elevated risk of adverse events in diabetic patients is closely linked to vulnerable plaque characteristics [27]. The COMBINE OCT-FFR trial demonstrated that even in the absence of flow limitation, the presence of OCT-detected thin-cap fibroatheroma significantly increased event risk in diabetic patients [28]. The PREVENT trial further indicated that for such non-flow-limiting vulnerable plaques, preventive PCI improved 2-year clinical outcomes regardless of diabetes status [13]. However, existing evidence primarily focuses on patients with either ischemic or nonischemic conditions, leaving the FFR_CT_ gray-zone population relatively understudied. Our analysis of this specific cohort indicates that, irrespective of diabetes status, the benefits of revascularization appear to be primarily associated with reductions in ischemic symptoms and unplanned interventions, while showing limited association with hard endpoints. The findings from our study are intended to inform future efforts toward developing more precise risk stratification and personalized treatment approaches for patients within this gray zone.

Restricted cubic spline analysis suggested that diabetes status may influence the relationship pattern between FFR_CT_ and MACE. A nonlinear relationship was observed in non-diabetic patients, whereas diabetic patients showed a trend of progressively increasing myocardial ischemia risk with declining FFR_CT_ values, which may be related to their predisposition to diffuse coronary disease and potential microvascular dysfunction [29]. The RCS analysis revealed an inverted U-shaped relationship between FFR_CT_ and MACE in non-diabetic patients. This pattern suggests that this intermediate-risk subgroup may face elevated risk due to clinical ambiguity leading to potential undertreatment, despite possibly having substantial plaque vulnerability or burden. Although revascularization benefits showed no significant interaction between subgroups, the optimal predictors appeared to differ: FFR_CT_ ≤ 0.75 might provide better predictive value in non-diabetic patients, whereas a coronary artery calcium score ≥ 300 could better identify high-risk patients in the diabetic population. These findings suggest that risk assessment in diabetic patients may need to consider overall atherosclerotic burden more comprehensively.

Several limitations should be considered when interpreting our findings. First, the retrospective design may introduce residual confounding and therefore requires validation in prospective studies. Second, detailed information on diabetes characteristics, including disease duration, classification, and glucose-lowering regimens, was not systematically collected, which may have affected outcome assessment. Given that the study was primarily designed to evaluate the fundamental association between diabetes status and outcomes among patients with borderline FFR_CT_, not the effects of specific subtypes or treatments such as disease duration, classification, or glucose-lowering regimens, it is acknowledged that this may have affected the outcome assessment. Third, the low incidence of hard endpoints may be explained by the predominance of chronic coronary syndrome in our population, potentially resulting from adequate risk factor control and frequent revascularization. Finally, our analysis did not include comprehensive plaque assessment, such as quantitative or qualitative morphological evaluation or perivascular adipose tissue analysis. Future prospective studies should incorporate these advanced plaque characteristics, potentially using artificial intelligence-assisted plaque analysis tools to enhance reproducibility and analytical efficiency.

## 5. Conclusions

Diabetic patients with borderline FFR_CT_ values demonstrated higher rates of invasive management, in whom FFR_CT_ further served as an independent predictor of MACE. Furthermore, while revascularization was associated with reduced MACE risk primarily through decreased unplanned revascularizations, this benefit did not extend to hard endpoints in the overall patient population, regardless of diabetes status.

## Figures and Tables

**Table 1:** Baseline characteristics according to diabetes status.

Characteristic	Non-Diabetes n = 1012	Diabetes n = 503	*p* Value
Age, yrs *	62 ± 10	63 ± 9	0.009
Sex, male	704 (69.6)	323 (64.2)	0.036
Follow-up, days ^†^	985 (507, 1141)	984 (501, 1149)	0.574
Hypertension	592 (58.5)	354 (70.4)	<0.001
Hyperlipidemia	623 (61.6)	347 (69.0)	0.005
Smoking	339 (33.5)	160 (31.8)	0.510
Family history of CVD	277 (27.4)	181 (36.0)	<0.001
Fast glucose, mmol/L ^†1^	5.36 (4.95, 5.90)	7.52(6.34, 8.91)	<0.001
HbA1c, % ^†2^	5.70 (5.50, 5.90)	7.00 (6.40, 7.70)	<0.001
Center			0.364
1	965 (95.4)	487 (96.8)	
2	21 (2.1)	6 (1.2)	
3	26 (2.6)	10 (2.0)	
Vessel location			0.210
LAD	692 (68.4)	322 (64.0)	
LCX	152 (15.0)	82 (16.3)	
RCA	168 (16.6)	99 (19.7)	
CACS ^†^	298 (82, 790)	534 (171, 1099)	<0.001
FFR_CT_ *	0.762 ± 0.024	0.758 ± 0.026	0.016
CAD-RADS			0.807
2	100 (9.9)	49 (9.7)	
3	551 (54.4)	266 (52.9)	
4	361 (35.7)	188 (37.4)	

Unless otherwise specified, data are numbers of patients, with percentages in parentheses. CVD = cardiovascular disease; LAD = left anterior descending artery; LCX = left circumflex artery; RCA = right coronary artery; CACS = coronary artery calcium score; FFR_CT_ = coronary CT angiography-derived fractional flow reserve; CAD-RADS = coronary artery disease reporting and data system. * Data are means ± SDs. ^†^ Numbers are medians, with IQRs in parentheses. ^1^ Data on fasting glucose were available for 63.8% (966/1515) of patients overall. ^2^ Data on HbA1c were available for 39.9% (604/1515) of patients overall.

**Table 2:** Comparative analysis of clinical outcomes based on diabetes status.

Outcome	All Patients (n = 1515)	Non-Diabetes (n = 1012)			Diabetes (n = 503)		
		OMT (n = 753)	REV (n = 259)	*p* Value	OMT (n = 348)	REV (n = 155)	*p* Value
MACE	117 (7.7%)	70 (9.3%)	13 (5.0%)	0.030	30 (8.6%)	4 (2.6%)	0.013
Secondary outcome	37 (2.4%)	22 (2.9%)	6 (2.3%)	0.609	7 (2.0%)	2 (1.3%)	0.728
All-cause death	27 (1.8%)	18 (2.4%)	5 (1.9%)	0.668	2 (0.6%)	2 (1.3%)	0.590
Non-fatal MI	10 (0.7%)	4 (0.5%)	1 (0.4%)	>0.999	5 (1.4%)	0 (0.0%)	0.330
Unplanned REV	89 (5.9%)	52 (6.9%)	8 (3.1%)	0.025	27 (7.8%)	2 (1.3%)	0.004

MACE = major adverse cardiovascular events; MI = myocardial infarction; OMT = optimal medical therapy; REV = revascularization. There were eight instances of unplanned revascularization following non-fatal MI, and one case of death following unplanned revascularization.

**Table 3:** Revascularization vs. medical therapy for MACE in gray-zone FFR_CT_: a Cox regression analysis stratified by diabetes.

Outcome	Non-Diabetes (n = 1012)				Diabetes (n = 503)			
	Univariate Analysis		Multivariate Analysis		Univariate Analysis		Multivariate Analysis	
	HR (95% CI)	*p* Value	HR (95% CI)	*p* Value	HR (95% CI)	*p* Value	HR (95% CI)	*p* Value
Age, yrs	1.02 (1.00, 1.04)	0.108			1.00 (0.96, 1.04)	0.928		
Sex, male	0.98 (0.61, 1.55)	0.916			1.56 (0.73, 3.35)	0.249		
Hypertension	1.16 (0.74, 1.80)	0.521			1.59 (0.69, 3.66)	0.273		
Hyperlipidemia	1.21 (0.77, 1.89)	0.411			0.95 (0.47, 1.93)	0.893		
Smoking	0.65 (0.40, 1.06)	0.086	0.69 (0.42, 1.12)	0.133	1.20 (0.60, 2.40)	0.602		
Family history of CVD	0.84 (0.51, 1.40)	0.512			0.62 (0.28, 1.36)	0.232		
Center								
1	—	—			—	—		
2	2.28 (0.71, 7.27)	0.164			3.50 (0.47, 25.87)	0.220		
3	1.20 (0.38, 3.82)	0.753			1.32 (0.18, 9.63)	0.787		
Vessel location								
LAD	—				—			
LCX	0.55 (0.25, 1.20)	0.133			1.68 (0.70, 4.04)	0.250	1.50 (0.62, 3.64)	0.366
RCA	1.38 (0.81, 2.34)	0.232			2.03 (0.93, 4.43)	0.076	2.16 (0.99, 4.73)	0.054
CACS								
0	—				—			
1–99	1.60 (0.60, 4.28)	0.350			1.97 (0.24, 16.40)	0.844		
100–299	0.84 (0.29, 2.42)	0.748			1.25 (0.14, 11.15)	0.529		
≥300	1.71 (0.68, 4.28)	0.256			1.88 (0.25, 13.89)	0.538		
FFR_CT_	0.95 (0.87, 1.03)	0.206			0.90 (0.80, 1.02)	0.098	0.87 (0.77, 0.99)	0.031
CAD-RADS								
2	—				—			
3	1.02 (0.48, 2.16)	0.968			1.28 (0.38, 4.31)	0.691		
4	1.13 (0.52, 2.47)	0.752			1.02 (0.29, 3.67)	0.972		
ICA	1.30 (0.85, 2.00)	0.229			1.33 (0.68, 2.60)	0.410		
REV	0.51 (0.28, 0.93)	0.028	0.53 (0.29, 0.96)	0.036	0.30 (0.11, 0.86)	0.025	0.25 (0.09, 0.71)	0.009

HR = hazard ratio; CVD = cardiovascular disease; LAD = left anterior descending artery; LCX = left circumflex artery; RCA = right coronary artery; CACS = coronary artery calcium score; FFR_CT_ = coronary CT angiography-derived fractional flow reserve; CAD-RADS = coronary artery disease reporting and data system; ICA = invasive coronary angiography; REV = revascularization.

## Data Availability

The datasets used and/or analyzed during the current study are available from the corresponding author on reasonable request.

## Supplementary Materials

The following supporting information can be downloaded at: https://www.mdpi.com/article/10.3390/jcdd13010011/s1, Figure S1: Interventional strategy distributions in diabetic and non-diabetic patients: a dual heatmap visualization; Figure S2: Comparison of ICA stenosis rates among patient groups stratified by diabetes and revascularization status; Figure S3: Kaplan-Meier curves for cause-specific survival analysis in diabetic and non-diabetic cohorts; Figure S4: Restricted cubic spline plots showing the association between FFR_CT_ values and MACE hazard, stratified by diabetes status; Figure S5: Covariate Balance Pre- and Post-Propensity Score Matching (SMD < 0.25 Threshold); Figure S6: Kaplan-Meier curves for the MACE in the propensity score-matched cohort; Figure S7: Sensitivity analysis excluding patients with unplanned revascularization within 30 days; Table S1: Baseline characteristics of participants stratified by diabetes and revascularization status; Table S2: Multivariable Cox regression analysis for MACE in the sensitivity analysis subgroup with complete glycemic data; Supplemental Methods.

## Abbreviations

DM	diabetes mellitus
CAD	coronary artery disease
FFR_CT_	CT-derived fractional flow reserve
CCTA	coronary CT angiography
ICA	invasive coronary angiography
PCI	percutaneous coronary intervention
OMT	optimal medical therapy
REV	revascularization
CABG	coronary artery bypass grafting
CAD-RADS	Coronary Artery Disease Reporting and Data System
CACS	coronary artery calcium score
MACE	major adverse cardiovascular event
