# Supplementary material for: Impact of Diabetes on Management and Outcomes in Patients with Borderline FFRCT
Source: J Cardiovasc Dev Dis. 2025 Dec 24;13(1):11. doi: 10.3390/jcdd13010011 (PMC12842064; doi:10.3390/jcdd13010011)
Supplement: Supplementary file 1 [file jcdd-13-00011-s001.zip › jcdd-4024823-supplementary.pdf]

Supplemental materials

Supplemental Table S1 Baseline characteristics of participants stratified by diabetes and revascularization status

Characteristic	Non-diabetes (n=1012)			Diabetes (n=503)		
	OMT (n=753)	REV (n=259)	P value	OMT (n=348)	REV (n=155)	P value
Age, years*	62±11	60±10	0.025	63±9	63±8	0.991
Sex, male	504 (66.9)	200 (77.2)	0.002	231 (66.4)	92 (59.4)	0.129
Follow-up, days†	984 (503, 1135)	1025 (548, 1174)	0.035	986 (501, 1149)	955 (501, 1147)	0.690
Hypertension	432 (57.4)	160 (61.8)	0.215	259 (74.4)	95 (61.3)	0.003
Hyperlipidemia	467 (62.0)	156 (60.2)	0.610	241 (69.3)	106 (68.4)	0.846
Smoking	236 (31.3)	103 (39.8)	0.013	105 (30.2)	55 (35.5)	0.238
Family history of CVD	217 (28.8)	60 (23.2)	0.078	125 (35.9)	56 (36.1)	0.964
Fast glucose, mmol/L †	5.39 (4.99, 5.89)	5.33 (4.91, 5.91)	0.356	7.57 (6.43, 8.80)	7.45 (6.23, 9.10)	0.935
HbA1c, %†	5.70 (5.50, 6.00)	5.70 (5.40, 5.90)	0.388	6.90 (6.40, 7.70)	7.00 (6.40, 7.90)	0.858
Center			0.462			>0.999
1	716 (95.1)	249 (96.1)		337 (96.8)	150 (96.8)	
2	15 (2.0)	6 (2.3)		4 (1.1)	2 (1.3)	
3	22 (2.9)	4 (1.5)		7 (2.0)	3 (1.9)	
Vessel location			0.486			0.158
LAD	511 (67.9)	181 (69.9)		222 (63.8)	100 (64.5)	

Characteristic	Non-diabetes (n=1012)			Diabetes (n=503)		
	OMT (n=753)	REV (n=259)	P value	OMT (n=348)	REV (n=155)	P value
LCX	119 (15.8)	33 (12.7)		63 (18.1)	19 (12.3)	
RCA	123 (16.3)	45 (17.4)		63 (18.1)	36 (23.2)	
CACS†	348 (96, 876)	190 (51, 634)	<0.001	574 (184, 1131)	468 (132, 1036)	0.133
FFR_{CT}*	0.766±0.022	0.749±0.026	<0.001	0.762±0.025	0.749±0.028	<0.001
CAD-RADS			<0.001			<0.001
2	89 (11.8)	11 (4.2)		42 (12.1)	7 (4.5)	
3	470 (62.4)	81 (31.3)		218 (62.6)	48 (31.0)	
4	194 (25.8)	167 (64.5)		88 (25.3)	100 (64.5)	

Unless otherwise specified, data are numbers of patients, with percentages in parentheses.

CVD=cardiovascular disease; LAD=left anterior descending artery; LCX=left circumflex artery; RCA=right coronary artery; CACS=coronary artery calcium score; FFR_{CT}=coronary CT angiography – derived fractional flow reserve; CAD-RADS=coronary artery disease reporting and data system.

* Data are means±SDs.

† Numbers are medians, with IQRs in parentheses.

Supplemental Table S2 Multivariable Cox regression analysis for MACE in the sensitivity analysis subgroup with complete glycemic data

Outcome	Non-diabetes (n=318)				Diabetes (n=251)			
	Univariate analysis		Multivariate analysis		Univariate analysis		Multivariate analysis	
	HR (95% CI)	p value	HR (95% CI)	P value	HR (95% CI)	p value	HR (95% CI)	P value
Age, yrs*	1.03 (0.98, 1.08)	0.228			1.00 (0.95, 1.05)	0.913		
Sex, male	2.39 (0.70, 8.12)	0.162			1.35 (0.47, 3.82)	0.577		
Hypertension	1.00 (0.41, 2.40)	0.991			1.51 (0.49, 4.62)	0.474		
Hyperlipidemia	1.08 (0.45, 2.62)	0.864			0.57 (0.22, 1.49)	0.255		
Smoking	0.61 (0.22, 1.66)	0.331			1.52 (0.58, 3.93)	0.393		
Family history of CVD	0.53 (0.16, 1.81)	0.312			0.59 (0.19, 1.80)	0.350		
Fast glucose, mmol/L†	0.71 (0.42, 1.21)	0.211			1.17 (1.01, 1.35)	0.031	1.14 (0.92, 1.40)	0.226
HbA1c, %†	0.97 (0.71, 1.32)	0.842			1.39 (0.98, 1.97)	0.067	1.14 (0.72, 1.79)	0.586
Vessel location								

LAD	—				—	
LCX	0.68 (0.15, 3.02)	0.612	0.64 (0.14, 2.85)	0.559	0.96 (0.21, 4.37)	0.956
RCA	2.46 (0.93, 6.47)	0.069	2.18 (0.82, 5.78)	0.118	1.42 (0.49, 4.16)	0.521
CACS†						
0	—				—	
1-99	—				1.53 (0.17, 13.69)	0.704
100-299	0.13 (0.02, 1.07)	0.058	0.13 (0.02, 1.11)	0.063	0.91 (0.08, 10.06)	0.940
≥300	0.86 (0.33, 2.24)	0.759	0.75 (0.28, 1.96)	0.552	1.06 (0.14, 8.24)	0.959
FFR_{CT}	0.98 (0.83, 1.16)	0.828			0.94 (0.79, 1.12)	0.486
CAD-RADS						
2	—				—	
3	1.15 (0.26, 5.20)	0.854			2.21 (0.29, 17.13)	0.448
4	0.76 (0.16, 3.59)	0.731			0.91 (0.11, 7.80)	0.932
ICA	3.23 (0.75, 13.86)	0.115			1.08 (0.38, 3.08)	0.880

REV	0.33 (0.12, 0.90)	0.031	0.33 (0.12, 0.91)	0.032	0.26 (0.07, 0.89)	0.032	0.26 (0.07, 0.89)	0.032

HR=hazard ratio; CVD=cardiovascular disease; LAD=left anterior descending artery; LCX=left circumflex artery; RCA=right coronary artery; CACS=coronary artery calcium score; FFR_{CT}=coronary CT angiography–derived fractional flow reserve; CAD-RADS=coronary artery disease reporting and data system; ICA=invasive coronary angiography; REV=revascularization.

Supplemental Figure S1 Interventional strategy distributions in diabetic and non-diabetic patients: a dual heatmap visualization

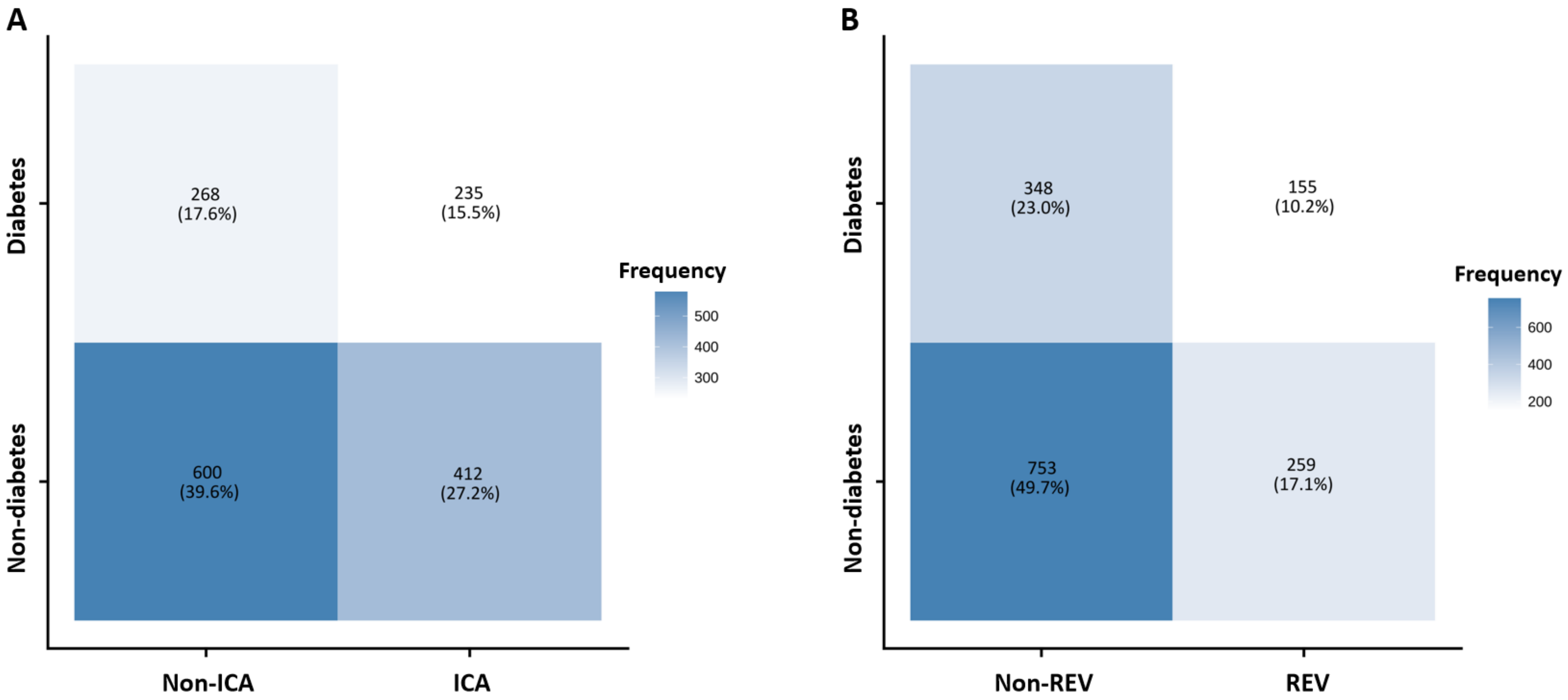

(A) Distribution of ICA rates; (B) Distribution of REV rates.

ICA=invasive coronary angiography; REV=revascularization.

Supplemental Figure S2 Comparison of ICA stenosis rates among patient groups stratified by diabetes and revascularization status

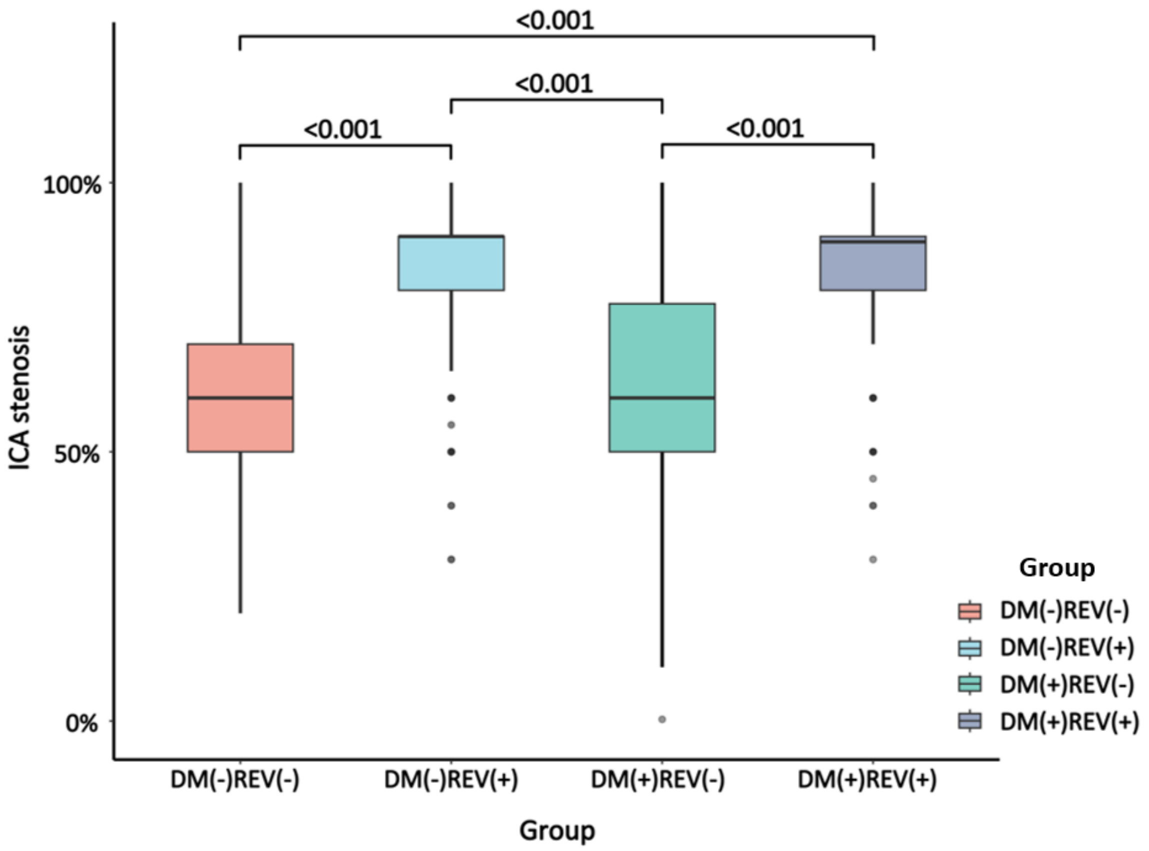

ICA=invasive coronary angiography; DM=diabetes mellitus; REV=revascularization.

Supplemental Figure S3 Kaplan-Meier curves for cause-specific survival analysis in diabetic and non-diabetic cohorts

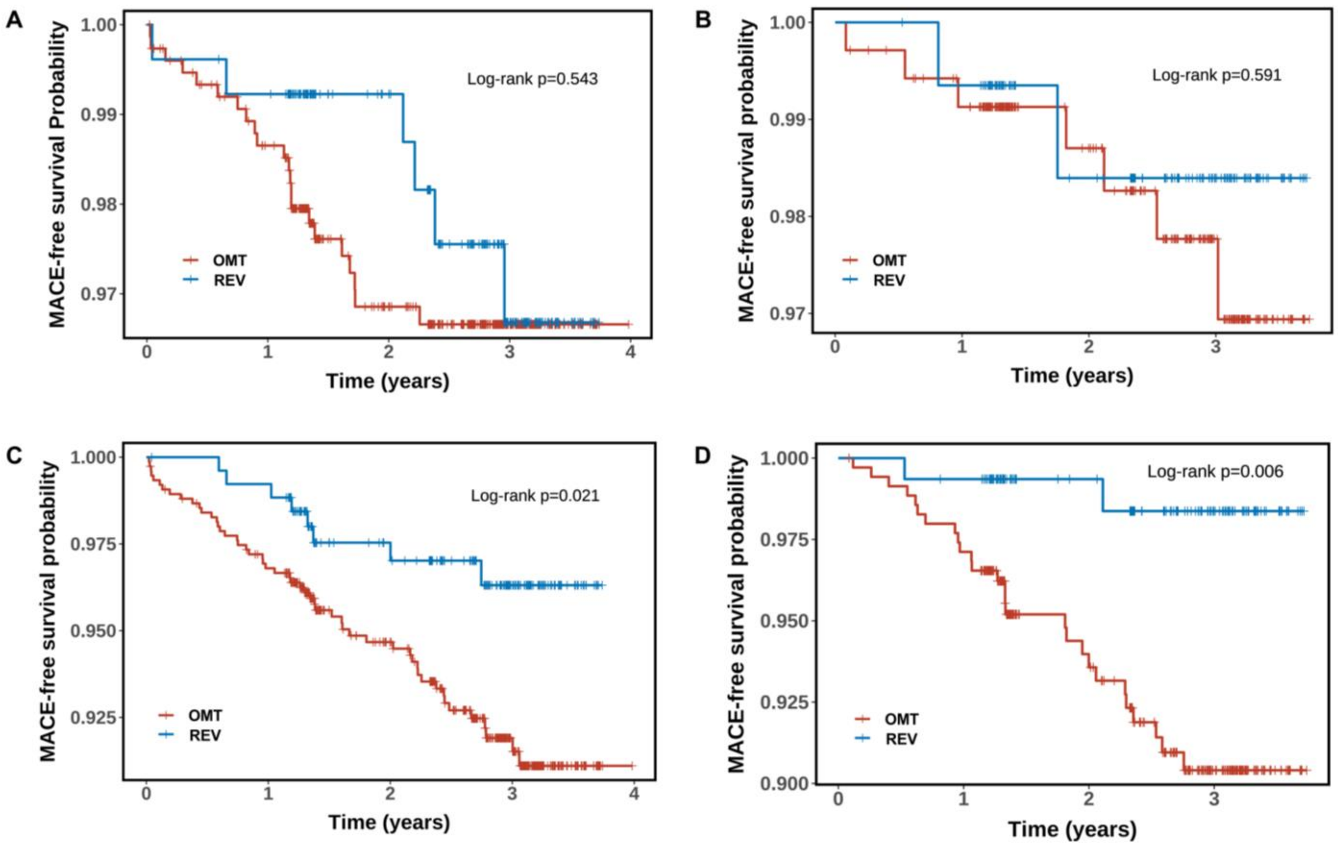

(A) Secondary outcome in non-diabetic group; (B) Secondary outcome in diabetic group; (C) Unplanned revascularization in non-diabetic group.

(D) Unplanned revascularization in diabetic group.

OMT=optimal medical therapy; REV=revascularization.

Supplemental Figure S4 Restricted cubic spline plots showing the association between FFR_{CT} values and MACE hazard, stratified by diabetes status

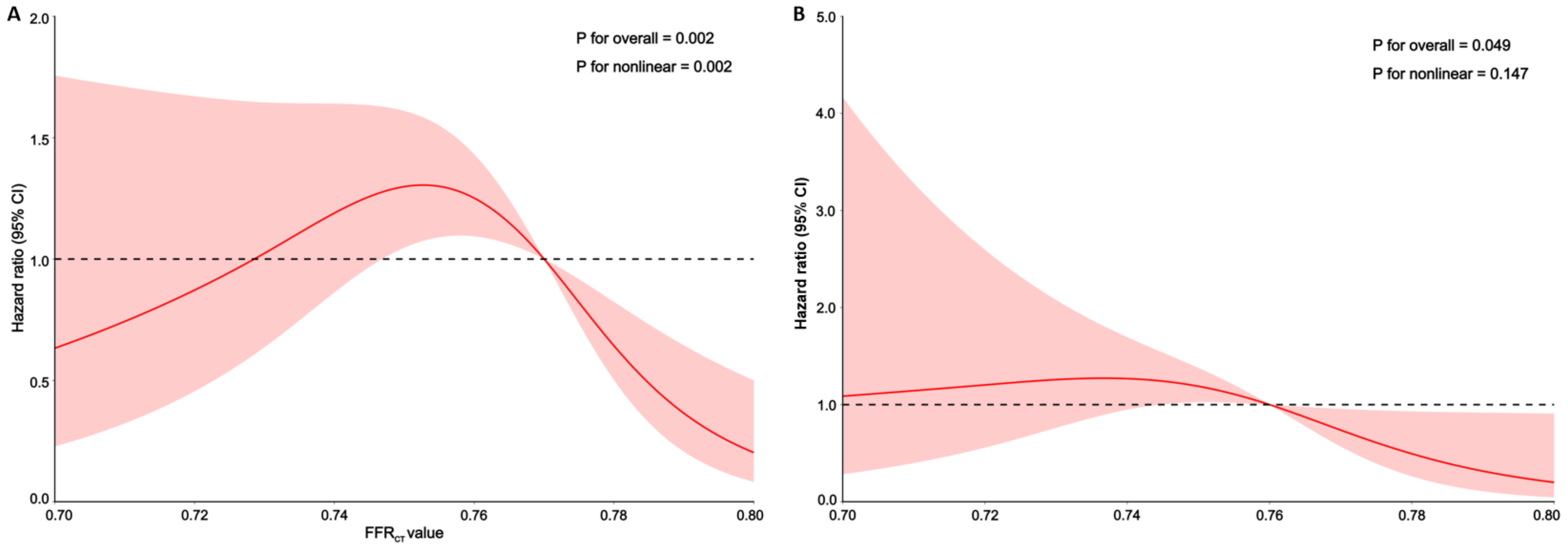

(A) Non-diabetic group; (B) Diabetic group.

HR=hazard ratio; $FFR_{CT=}$coronary CT angiography-derived fractional flow reserve.

Supplementary Figure S5 Covariate Balance Pre- and Post-Propensity Score Matching (SMD < 0.25 Threshold)

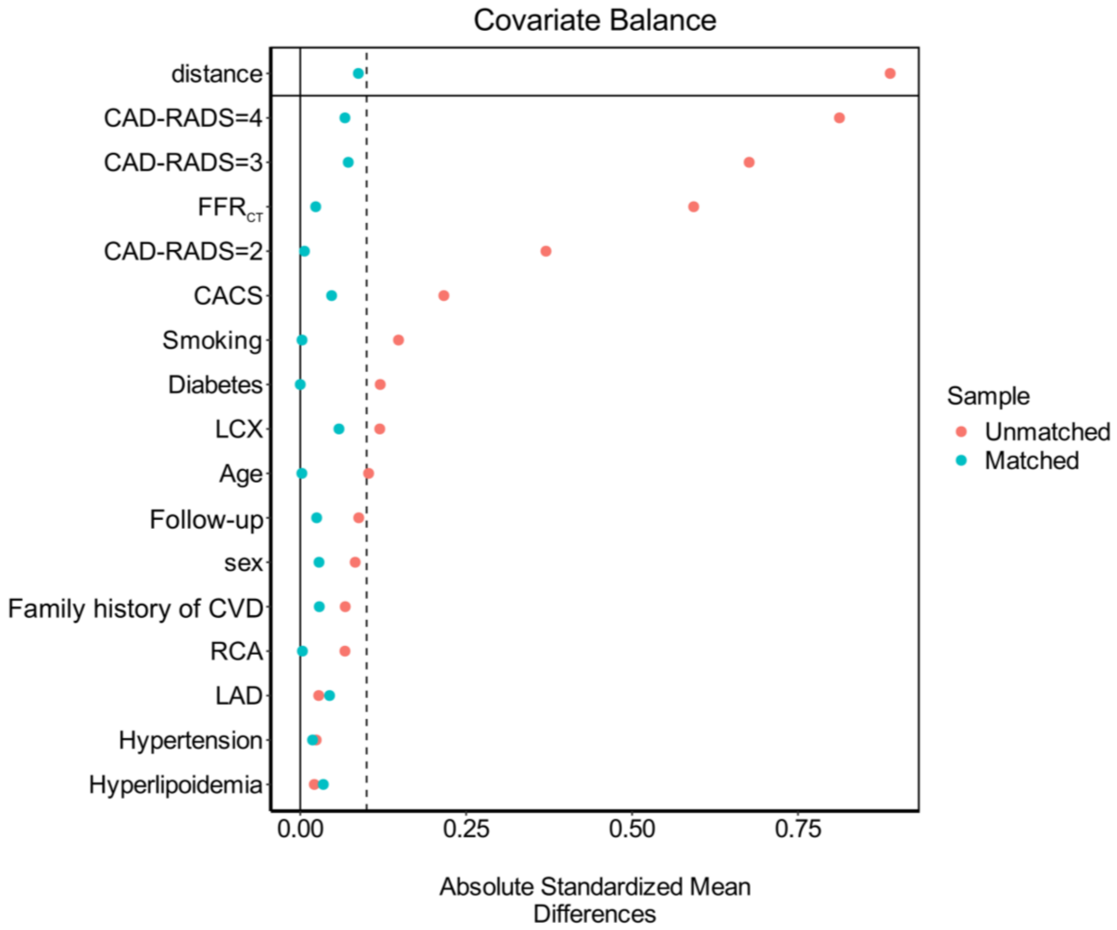

CAD-RADS=coronary artery disease reporting and data system; FFR_{CT}=coronary CT angi-ography﹣derived fractional flow reserve; CACS=coronary artery calcium score; LCX=left circumflex artery; CVD=cardiovascular disease; RCA=right coronary artery; LAD=left anterior descending artery.

Supplementary Figure S6 Kaplan-Meier curves for the MACE in the propensity score-matched cohort

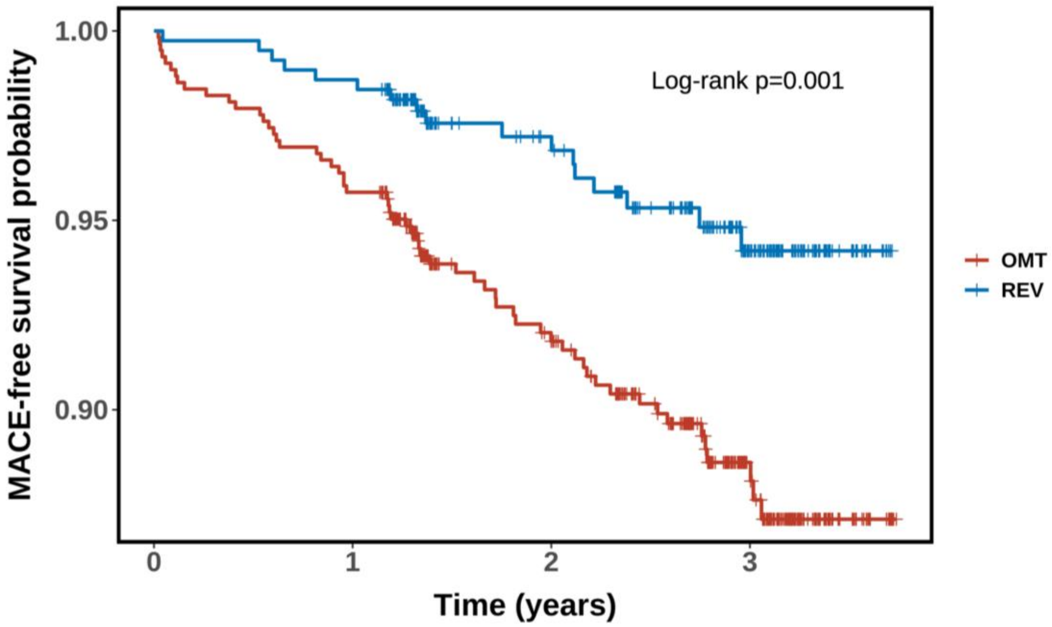

		0	1	2	3
OMT	At Risk	587	562	402	180
	Events	0	25	44	56
REV	At Risk	388	383	268	134
	Events	0	5	10	17

OMT=optimal medical therapy; REV=revascularization.

Supplementary Figure S7 Sensitivity analysis excluding patients with unplanned revascularization within 30 days

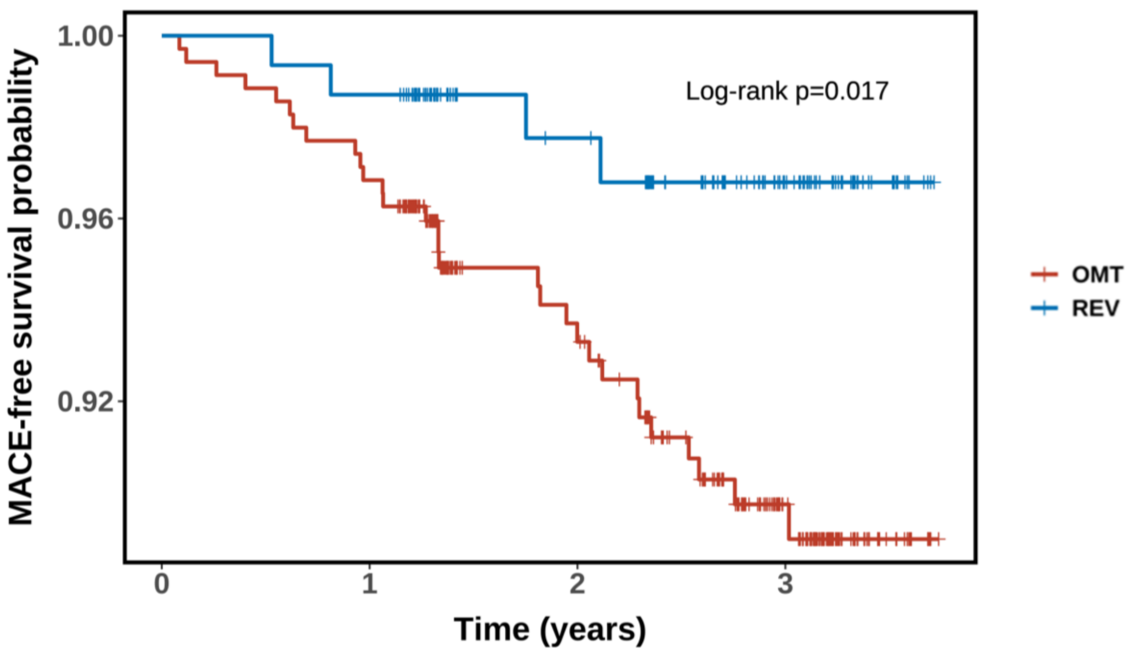

	0	1	2	3
**OMT**				
At Risk	348	337	230	119
Events	0	11	21	29
**REV**				
At Risk	155	153	102	49
Events	0	2	3	4

OMT=optimal medical therapy; REV=revascularization.

Supplemental Methods

CCTA Protocols

1)** hospital: All patients underwent CCTA using one of the following CT platforms: a dual-source CT scanner (Somatom Definition Flash, Siemens Healthineers), a 256-section wide-detector CT scanner (Revolution CT, GE Healthcare), or a 320-detector row CT scanner (Aquilion One, Canon Corporation). Prospective electrocardiographic gating was applied for all scans using the following R-R interval ranges: 25%–55% for patients with heart rates (HR) >80 bpm, 30%–80% for HR <80 bpm, and 65%–85% for HR <65 bpm. Heart rate–controlling medications were not administered before CCTA, as patients with high HR (>100 bpm) or arrhythmias were advised to consult a cardiologist for further evaluation. The CT platforms used provided enhanced temporal resolution, allowing for diagnostic image quality even at higher heart rates. Additionally, avoiding pharmacologic HR control minimized the risk of adverse drug effects and reduced waiting times for CCTA procedures. The scanning range extended from 1.0 cm below the tracheal carina to 1.5 cm below the heart. A real-time bolus-tracking technique was used to automatically trigger image acquisition, with the region of interest placed in the ascending aorta. During image acquisition, 0.8 mL/kg of contrast agent (Iohexol 350, GE Ltd., USA) was injected at a flow rate of 4.0–5.0 mL/s, followed by a 30 mL saline flush. The scan parameters were as follows:

a. Dual-source CT scanner: Rotation time, 0.28 s; pixel matrix, 512×512; collimation, 2×64×0.6 mm; tube voltage, 100 or 120 kV with automatic tube current selection; slice thickness, 0.6 mm; reconstruction increment, 0.6 mm. Raw data were reconstructed using an iterative reconstruction (IR) algorithm (SAFIRE, Siemens Healthineers) with a strength level of 3.

b. 256-section wide-detector CT scanner: Rotation time, 0.28 s; pixel matrix, 512×512; collimation, 256×0.625 mm; tube voltage, 100 or 120 kV with Smart mA modulation; slice thickness, 0.625 mm; reconstruction increment, 0.625 mm. Raw data were reconstructed using the ASiR-V™ IR algorithm (GE Healthcare) at 60% strength.

c. 320-detector row CT scanner: Rotation time, 0.35 s; pixel matrix, 512×512;

collimation, 320×0.5 mm; tube voltage, 100 or 120 kV; tube current, 200–400 mA; slice thickness, 0.5 mm; reconstruction increment, 0.5 mm. Raw data were reconstructed using a medium soft-tissue convolution kernel (I26f/Bv40).

2) ** Hospital: Image acquisition was performed using second- or third-generation dual-source CT systems (SOMATOM Definition Flash or SOMATOM Force; Siemens Healthineers). Coronary CT angiography (CCTA) was conducted using contrast-enhanced, prospectively ECG-gated protocols with tube voltage ranging from 70 to 120 kV and tube current between 200 and 650 mAs, adjusted according to patient body size. Contrast enhancement was achieved by injecting 50–80 mL of iopromide at a rate of 4–6 mL/s, followed by a 30 mL saline bolus chaser. Beta-blockers and nitroglycerin were administered at the discretion of the attending physician. Image reconstruction was performed using a section thickness of 0.75 mm, a reconstruction increment of 0.5 mm, and a smooth convolution kernel (I26f or Bv40).

3) ** Hospital: CCTA examinations were performed using a 256-section wide-detector CT scanner (Brilliance iCT, Philips; or Revolution CT, GE Healthcare) in axial mode. Tube voltage was adjusted between 70 and 120 kV, and tube current was modulated between 200 and 600 mA based on patient characteristics. Images were acquired at a slice thickness of 0.5–0.75 mm and reconstructed using iterative reconstruction algorithms. Contrast enhancement involved administering 60–80 mL of Iohexol at a flow rate of 4–6 mL/s, followed by a 30 mL saline flush. Electrocardiographic synchronization was achieved through prospective triggering at 70–80% of the R-R interval. The spatial and temporal resolutions were 0.23 mm isotropic and 75 ms, respectively. The total radiation dose ranged from 1 to 4 mSv.
